# Impact of DRG payment reform on revenue and expenditure structure of public hospitals in China: evidence from difference-in-differences analysis

**DOI:** 10.3389/fpubh.2025.1725444

**Published:** 2026-01-22

**Authors:** Xiaofei Chu, Yanlei Hou

**Affiliations:** School of Business, Henan University of Science and Technology, Luoyang, Henan, China

**Keywords:** DRG payment reform, revenue and expenditure structure, difference-in-differences, propensity score matching, resource allocation efficiency, public hospitals

## Abstract

**Background:**

Diagnosis-related group (DRG) payment reform has been widely promoted in China to curb the rapid growth of health expenditure, but its effects on the revenue and expenditure structure and resource allocation efficiency of public hospitals remain unclear.

**Methods:**

This study used panel data from 48 tertiary public hospitals in China from 2017 to 2023 and applied a quasi-experimental design combining propensity score matching and a difference-in-differences approach to compare hospitals implementing DRG payment reform with non-DRG hospitals.

**Results:**

We examined changes in the composition of hospital revenues and expenditures, concentration indices of income and costs, and efficiency indicators such as the revenue-to-cost ratio and per capita revenue efficiency. Among DRG-paying hospitals, the share of medical service revenue increased from 42.6% to 48.9%, while the shares of drug and material revenue decreased from 29.1 to 24.3% and from 22.8% to 20.4%, respectively; personnel expenditure rose from 35.2% to 39.8%, whereas drug expenditure declined from 28.5% to 23.7%. The income concentration index increased from 0.342 to 0.428 and the expenditure concentration index from 0.356 to 0.441, indicating reduced cross-hospital variability and greater standardization of revenue and expenditure patterns. The revenue-to-cost ratio improved from 1.082 to 1.136, and per capita revenue efficiency increased by 11.0%, suggesting a significant enhancement in hospital resource allocation efficiency.

**Conclusion:**

DRG payment reform has optimized the revenue and expenditure structure of Chinese tertiary public hospitals, reduced dependence on drugs and materials, strengthened incentives for investment in human resources, and supported the shift from scale expansion to quality- and efficiency-oriented development.

## 1 Introduction

In recent years, health expenditure in China has maintained a continuous and rapid growth trend, which has become a prominent challenge in the field of healthcare. Official statistics show that between 2017 and 2022, the total health expenditure in China registered an average annual growth rate of approximately 10%, with its share of gross domestic product (GDP) rising from 6.36% to 7.05% and further approaching 7.2% in 2023 (1, 2). The rapid expansion of health expenditure is underpinned by multiple intertwined drivers, encompassing population aging, changes in the disease spectrum, advancements in medical technology, and an increase in the demand for medical services (3). Not only does government health expenditure contribute to economic growth, but it also exerts an upward pressure on the overall level of medical costs (4). With the continuous expansion of China’s public health system, the relationship between the input and output of medical resources has become increasingly complex (5). As a pivotal socioeconomic factor, the urbanization process has had a significant impact on the growth of health expenditure. The improvement of the urbanization level has brought about fundamental changes in the utilization pattern of medical services (6). In China, the healthcare financing system operates as a mixed model integrating government budget allocations, social health insurance contributions, and individual out-of-pocket payments, distinct from the pure tax-funded Beveridge model. Government and social insurance funds together underpin the majority of health expenditure, while out-of-pocket payments still account for a non-negligible share of total spending. The rapid growth of health expenditure not only exacerbates the financial burden on the government but also increases the economic strain on patients. Consequently, there is an urgent imperative to curb the unreasonable growth of health costs and optimize the efficiency of medical resource allocation through the reform of healthcare payment systems.

The traditional fee-for-service (FFS) payment model, while incentivizing the provision of medical services, also gives rise to systematic problems such as overtreatment and resource waste, prompting countries to seek more effective payment system reform initiatives (7). Empirical studies have demonstrated that different payment mechanisms exert significantly heterogeneous impacts on physicians’ clinical practice behaviors. The FFS model is prone to inducing moral hazard, with supplier-induced demand being a typical manifestation (8). Although some perspectives argue that FFS payment is not the main cause of the health expenditure growth (9), its limitations in cost control and the efficiency of resource allocation have been widely recognized. As a pivotal innovation designed to address the drawbacks of the Fee-for-Service (FFS) system, the Diagnosis-Related Groups (DRG) payment system has evolved into a globally recognized medical payment mechanism since its successful implementation in the US Medicare system in the 1980s (10). During its promotion and implementation across European countries, this system has exhibited remarkable cost control effects. Implementation experiences derived from diverse healthcare systems consistently converge on shared objectives, including enhanced transparency, improved operational efficiency, and optimized service quality (11). After more than three decades of development and refinement, the DRG payment system has become a pivotal orientation for the reform of modern medical payment systems.

International practices have demonstrated that the DRG payment system exhibits strong adaptability and favorable reform effects across different healthcare systems worldwide. After the implementation of the DRG reform in the US Medicare system, significant progress was achieved in curbing the growth of inpatient healthcare costs and optimizing the allocation of medical resources. However, it also confronts non-negligible challenges, such as the adequacy of risk adjustment and the fairness of payment (12–14). The implementation experience from the mandatory DRG payment system in Asian countries like South Korea shows that this payment mechanism can effectively optimize the utilization pattern of inpatient medical services. Systematic studies have confirmed that, compared with the cost-based payment model, DRG payment has distinct advantages in curbing the growth of inpatient medical costs (14).

Since 2011, China has launched pilot projects of DRG payment reform in selected regions, accumulating implementation experience through a phased dual-track reform approach (15). Empirical studies conducted in pilot areas such as Beijing have shown that the DRG payment reform has effectively reduced hospital expenditures and patients’ out - of - pocket expenses (16, 17). The pilot experience provides important policy references and practical foundations for the nationwide promotion of the DRG payment system.

Although rich practical experience has been accumulated in DRG payment reforms both at home and abroad, existing empirical studies remain relatively limited in terms of explicit theoretical framing and methodological rigor. From a health economics and organizational perspective, DRG-based prospective payment can be understood within a principal–agent framework, in which payers (such as governments and social health insurance funds) act as principals and hospitals as agents whose behavior is shaped by the financial incentives embedded in payment contracts (18). However, many DRG studies do not explicitly draw on such theoretical frameworks when analyzing how payment reform affects provider behavior, and instead mainly focus on descriptive changes in utilization or costs. Current studies mainly focus on the impacts of the DRG payment system on aspects such as medical service quality, service efficiency, and capacity utilization (19), while the systematic analysis of changes in hospitals’ revenue and expenditure structures is relatively insufficient. In China, DRG pricing and payment policies are still in the stage of exploration and improvement, and there is limited empirical research evidence in this regard (20). Most of the existing literature uses descriptive analysis methods and lacks empirical research designs based on strict causal inference. Furthermore, the complex changes in the external environment confronting the reform of the medical payment system, such as issues in the drug supply chain and the impact of public health emergencies (21, 22), have further heightened the difficulty of accurately assessing the net effect of the DRG reform.

This study aims to systematically evaluate the impact of the DRG payment reform on the revenue and expenditure structure of public hospitals in China. This study selects the tertiary-level hospitals, which are the most representative in the DRG payment reform, as the analysis objects. These hospitals have the most complete financial data. A quasi-experimental framework is constructed using the difference-in-differences (DiD) method. By comparing and analyzing the changes in the hospital’s revenue composition, expenditure structure, and resource allocation efficiency before and after the DRG reform, the causal effect of the reform is identified. The research results will provide empirical evidence for deepening the reform of the medical payment system, support the optimization and improvement of the DRG payment policy, and promote the establishment of a more reasonable and effective medical payment system. Furthermore, this study will contribute to the high-quality development of public hospitals and the sustainable advancement of the medical and health sector in China.

## 2 Materials and methods

### 2.1 Research design

This study uses a quasi-experimental design to evaluate the impact of the DRG payment reform on the revenue and expenditure structure of public hospitals (23). The study constructs a comparative analysis framework for the DRG payment group and the non-DRG payment group. Taking the implementation of the DRG reform in January 2019 as the time cutoff point, the hospitals participating in the reform are defined as the intervention group, while those not participating in the reform are defined as the control group. By comparing the differential changes in the revenue and expenditure structure indicators of the two groups of hospitals before and after the reform, the net effect of the DRG payment reform is identified.

The study design strictly adheres to the methodological requirements and bias risk assessment standards of quasi-experimental research, thereby ensuring the internal and external validity of the findings (24). This research design can effectively control for confounding factors such as time trends and hospital fixed effects, providing a reliable basis for causal inference in policy effect evaluation. To reduce the impact of selection bias on the research results, the study used the propensity score matching (PSM) method to balance the covariate distributions between the intervention group and the control group. The calculation of the propensity score was based on key variables such as hospital scale, number of beds, medical staff allocation, and patient composition. The probability of each hospital participating in the DRG payment reform was estimated through a Logistic regression model. In the matching process, a 1:1 nearest neighbor matching method was used in the matching process, and the matching tolerance was set at 0.05 to ensure the matching quality and sample balance.

The study conducted an economic analysis from the perspective of hospital managers, focusing on the changes in the hospitals’ direct revenues and costs, including medical service income, drug income, material income, and the corresponding adjustment of the expenditure structure. The standard deviation (SD) is calculated to measure the dispersion of the revenue and expenditure distribution, and the difference-in-differences (DiD) value is used to qualify and compare the differences in revenue and expenditure changes between the two groups. The concentration index is used to evaluate the uniformity of the revenue and expenditure distribution, where a value close to 0 indicates a relatively uniform distribution. The study uses the difference-in-differences (DiD) model to evaluate the causal effect of the DRG payment reform. This model can control for the hospital fixed effects that do not change over time and the time trend effects that do not vary across hospitals. To verify the core assumption of the DiD method, the study conducts a parallel trend test to confirm that the intervention group and the control group had similar development trends in revenue and expenditure indicators before the reform.

Compared with alternative quasi-experimental methods, DiD is particularly well-suited to our study setting. Unlike the synthetic control method, which is typically applied to a single or a few treated units and requires constructing weighted combinations of control units to replicate the treated unit’s pre-intervention trajectory (25, 26), DiD is more appropriate when there are multiple treated units (24 DRG-paying hospitals in our case) and when the parallel trends assumption is plausible (27). Unlike regression discontinuity designs, which require treatment assignment based on a clear threshold or cutoff point in a continuous variable (28), our DRG reform was implemented at the hospital level based on policy eligibility criteria rather than a sharp discontinuity, making DiD the more natural choice. Recent methodological work has further emphasized the advantages of DiD designs for health policy evaluation, provided that the parallel trends assumption is appropriately assessed through pre-reform trend testing (29).

### 2.2 Data sources and sample selection

Small public hospitals generally face problems such as non-public or missing data and lagging reforms. In China’s hospital accreditation system, Class-III Grade-A hospitals are large tertiary public hospitals with the highest accreditation level. They provide high-level specialized medical services and undertake advanced teaching and research tasks, and are broadly comparable to tertiary teaching hospitals in other health systems. Therefore, this study takes China’s Class-III Grade-A public hospitals as the research object to evaluate the causal effects of the DRG reform. The research data were obtained from audited annual financial statements and routine operational reports submitted by Class-III Grade-A public hospitals to the municipal or provincial health commissions and healthcare security bureaus, as well as from DRG reform monitoring databases maintained by these authorities between January 2017 and December 2023. All data were de-identified by the data-providing institutions before being made available to the research team. We selected 2017 as the starting year because hospital financial reporting and DRG-related cost accounting standards had been largely standardized by that time, and no major payment reforms affecting inpatient revenue and expenditure structures were implemented in the study regions between 2017 and the launch of the DRG reform in 2019, which provides a relatively stable pre-reform baseline for the difference-in-differences analysis. The sample hospitals are distributed in the eastern, central, and western regions, covering general hospitals and specialized hospitals, and are well-representative.

The DRG payment reform was officially implemented in some hospitals in January 2019, covering major departments such as general surgery, cardiothoracic surgery, neurosurgery, and urology. The sample selection follows the principles of scientific sampling methodology to ensure the adequacy and representativeness of the sample size (30). Control hospitals were Class-III Grade-A public hospitals located in non-pilot cities that were not included among the 30 national DRG pilot cities designated in 2019, and therefore continued to be reimbursed mainly under pre-existing fee-for-service–based payment arrangements throughout 2017–2023. DRG-paying hospitals were selected from the national pilot cities if they met the following criteria: (1) Class-III Grade-A public hospitals that had been in continuous operation from 2017 to 2023; (2) complete and consistently recorded annual financial and operational data for all study years; and (3) no major organizational changes such as mergers, splits or ownership restructuring during the observation period. The exclusion criteria include: hospitals with more than 10% of data missing; hospitals that underwent major restructuring or mergers during the study period; and hospitals affected by other major policy interventions. For each DRG-paying hospital, we selected one Class-III Grade-A public hospital in a non-pilot city as a control, matched in terms of geographic region (eastern, central, or western China), hospital type (general vs. specialized), and baseline scale (number of beds and total revenue). This sampling strategy ensured that the 48 hospitals cover different macro-regions, include both general and specialized tertiary hospitals, and reflect typical characteristics of large public hospitals in China, thereby enhancing the representativeness of the sample. The final sample included in the analysis consists of 24 hospitals implementing the DRG reform and 24 control hospitals, totaling 84 months of panel data from 48 hospitals.

### 2.3 Variable definitions

The indicators of hospital income structure include four dimensions: medical service income, drug income, material income, and other income. Medical service income includes income items that reflect the technical labor value of medical staff, such as diagnosis and treatment fees, surgical fees, nursing fees, and bed fees. Drug income comprises the sales income from Western medicines, Chinese patent medicines, Chinese herbal medicines, and other pharmaceutical products. Material income refers to the sales income of medical materials such as medical devices and medical consumables. Other income covers inspection fees and other medical service income. The reform of the medical system has exerted a significant impact on the income structure of public hospitals, and changes in income composition serve as an important indicator for evaluating the effectiveness of the payment system reform (30).

The indicators for hospitals’ expenditure structure encompass core cost components such as personnel expenditure, drug expenditure, material expenditure, and equipment depreciation. Personnel expenditure includes human resource costs such as salaries, bonuses, social insurance, and housing provident fund for medical staff. Drug expenditures refer to the costs of drug procurement, and material expenditures refer to the costs of medical material procurement. Equipment depreciation includes the depreciation expenses of medical equipment. The establishment of a comprehensive financial indicator system facilitates the holistic evaluation of hospitals’ operational efficiency and cost-containment effectiveness (31).

Control variables include factors such as hospital scale, number of beds, number of medical staff, average age of patients, gender composition, and disease severity. Under the case-based payment system, the behavioral adjustment of hospitals is affected by various factors. Relevant confounding variables need to be controlled to accurately identify the policy effect (32).

### 2.4 Statistical methods

To reduce the confounding bias caused by differences in hospital characteristics, the study uses the propensity score matching method to balance the covariate distribution between the intervention group and the control group (33). The propensity score of each hospital was calculated using the Logistic regression model. The matching variables included key covariates such as hospital scale, number of beds, medical staff allocation, patient composition, and regional economic level. One-to-one nearest neighbor matching without replacement was adopted, and the matching tolerance was set at 0.05 to ensure matching accuracy. After matching, the standardized differences and *t*-tests were used to evaluate the balance of the matching variables. A standardized difference of less than 0.1 indicated good balance between variables. Meanwhile, a Love plot was generated to visualize the distribution of covariables before and after matching, thereby further validating the matching effectiveness.

To evaluate the causal impact of the DRG payment reform on the revenue and expenditure structure of hospitals, the study adopted the difference-in-differences model (34). We chose a difference-in-differences (DiD) design (Equation 1) because it combines before-and-after and treatment–control comparisons in a single framework and is widely regarded as a robust quasi-experimental method for policy evaluation when randomized experiments are not feasible. By comparing changes over time between DRG-paying and non-DRG hospitals, the DiD approach removes time-invariant differences between groups and common time shocks that affect all hospitals, thereby reducing confounding that would bias simple before-and-after comparisons or cross-sectional regression adjustments (35). Recent methodological work has further emphasized the advantages of DiD designs for health policy evaluation, provided that the parallel trends assumption is plausible and appropriately assessed (29). The model estimates the net effect of the reform by comparing the changes in the revenue and expenditure structures between the DRG payment group and the non-DRG payment group before and after the reform. The specific model settings are as follows:


(1) $$Y_{\mathrm{it}}=\alpha +\beta_{1}{\text{Post}}_{t}+\beta_{2}{\mathrm{DRG}}_{i}+\beta_{3}({\text{Post}}_{t}\times {\mathrm{DRG}}_{i})+\gamma X_{\mathrm{it}}+\varepsilon_{\mathrm{it}}$$

Where $Y_{\mathrm{it}}$ denotes the income and expenditure structure indicator of hospitals i at time t ; $\mathrm{Pos}t_{t}$ is a time dummy variable (after DRG payment reform = 1, otherwise = 0); $\mathrm{DR}G_{i}$ is a group dummy variable (DRG-paying hospitals = 1, otherwise = 0); $X_{\mathrm{it}}$ is a vector of control variables; and $\varepsilon_{\mathrm{it}}$ is a random error term. The interaction term coefficient $\beta_{3}$ captures the average treatment effect of DRG payment reform.

To ensure the robustness of the model, this study conducts a placebo test and a parallel trend test. Specifically, the placebo test verifies model robustness by randomly assigning a dummy reform time point to years when the DRG payment reform was not implemented, thereby examining whether spurious significant changes in costs emerge. A parallel trend test is also conducted using pre-reform data to confirm that the revenue and expenditure trends of the DRG-paying and non-DRG-paying groups remain parallel in the pre-reform period to validate the validity of the DiD model assumptions.

The study employed the concentration index to assess the consistency of income and expenditure distribution (36, 37). A concentration index approaching 0 indicates a relatively uniform distribution of revenues and expenditures. The impact of DRG payment reform on cost equity and consistency was evaluated by analyzing the changes in the concentration index in different years. The Kruskal–Wallis test was used to analyze the significance of changes in the concentration index.

### 2.5 Cost standardization process

To ensure the comparability and accuracy of revenue and expenditure data (38), all revenue and expenditure indicators in this study were adjusted to the 2019 constant price level (Equation 2). The adjustment was carried out based on the consumer price index (CPI) released by the National Bureau of Statistics. As an important indicator reflecting changes in the price level of consumer goods and services, the CPI can better reflect changes in the level of inflation. The reason for choosing 2019 as the base period is that this year is the starting point for the implementation of the DRG payment reform, and using it as a benchmark can provide a more intuitive view of the actual changes before and after the reform. Ideally, a health care–specific price index would be used to deflate hospital revenues and expenditures, because health care prices often rise faster than general consumer prices. However, consistent medical price indices are not publicly available for all provinces and years covered by our study. We therefore use the national CPI as a pragmatic approximation and acknowledge that this approach may lead to conservative estimates of the real growth in health care costs. All monetary values are adjusted to a uniform price level to ensure the accuracy and credibility of the study results.

The calculation formula is:


(2) $$\text{Adjusted Cost}=\text{Original Cost}\times \frac{{\mathrm{CPI}}_{2019}}{{\mathrm{CPI}}_{x}}$$

## 3 Results

### 3.1 Sample characterization

A total of 48 tertiary public hospitals were finally included in this study, including 24 DRG payment reform hospitals and 24 control hospitals. The observation period spanned from January 2017 to December 2023, totaling 84 months. The geographical distribution of the sample hospitals was balanced, with 52.1% of hospitals in the eastern region, 31.3% in the central region, and 16.6% in the western region. The composition of hospital types was well represented, with 75.0% of general hospitals and 25.0% of specialized hospitals.

The basic characteristics of the sample hospitals are shown in Table 1. Specifically, the average number of beds in hospitals implementing the DRG payment reform (intervention group) was 1,247.3, while that in the control group hospitals was 1,189.7, with no statistically significant difference observed between the two groups (*p* = 0.342). In terms of medical staffing, the average number of medical staff in reform hospitals was 892.5, and 847.2 in control hospitals (*p* = 0.287). Patient composition characteristics showed that the average age of patients in the reform hospitals was 52.4 years and 51.8 years in the control hospitals (*p* = 0.456); the average length of hospital stay was 8.9 and 9.2 days, respectively (*p* = 0.523).

**Table 1:** Comparison of basic characteristics of the sample hospitals.

Characteristics Indicators	DRG reform group (*N* = 24)	Control group (*N* = 24)	*t*-value	*p*-value
Number of beds	1247.3 ± 235.6	1189.7 ± 198.4	0.968	0.342
Number of medical staff (persons)	892.5 ± 156.3	847.2 ± 142.7	1.078	0.287
Average age of patients (years)	52.4 ± 8.9	51.8 ± 9.2	0.753	0.456
Average hospitalization (days)	8.9 ± 2.1	9.2 ± 2.3	−0.645	0.523
Annual outpatient volume (10,000 visits)	78.6 ± 15.2	81.3 ± 17.8	−0.572	0.571
Annual hospitalization (10,000)	4.2 ± 0.8	4.1 ± 0.9	0.425	0.673

**Figure 1:**
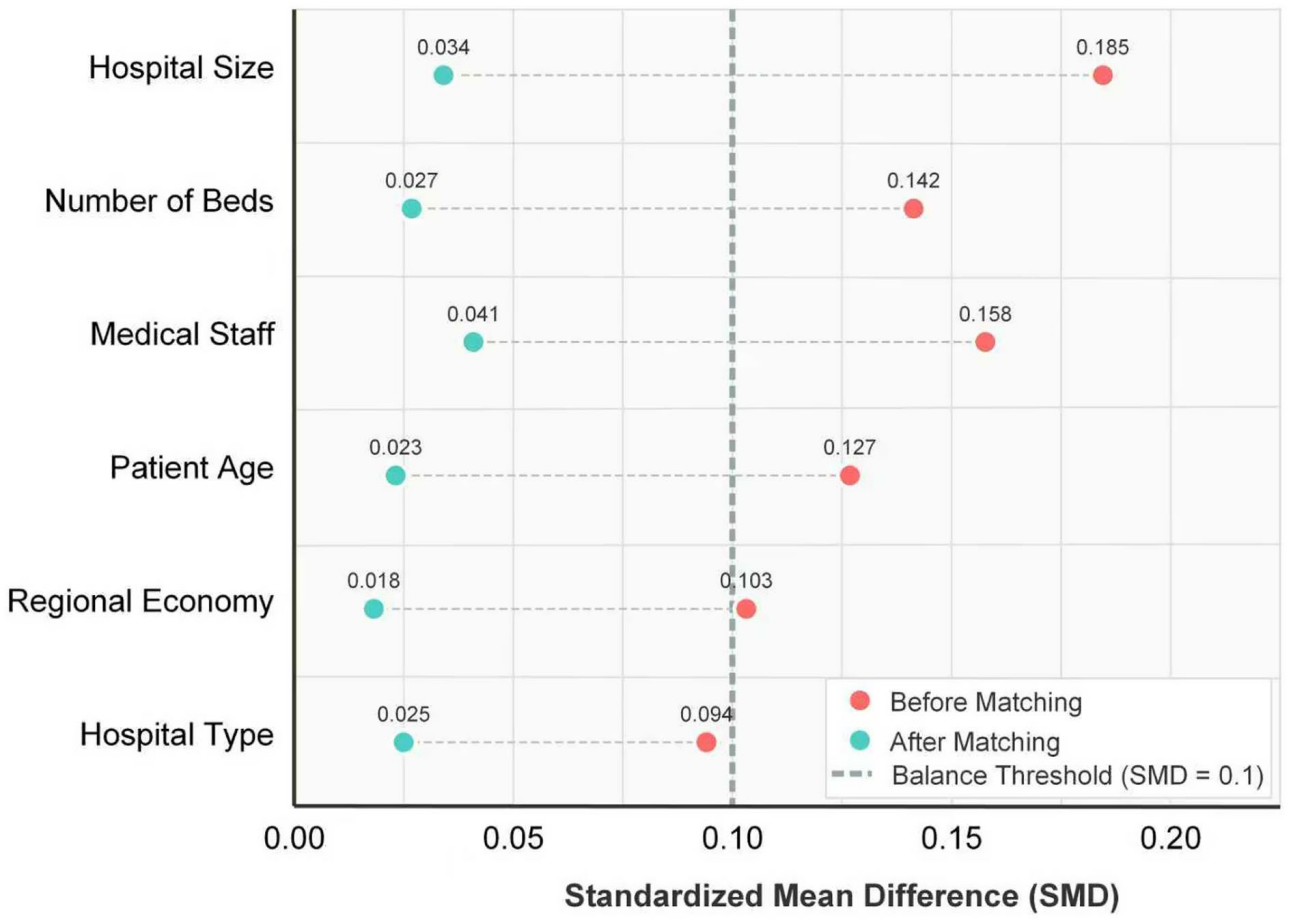
Standardized mean differences before and after PSM.

The balance of covariates after propensity score matching is shown in Table 2. The standardized mean differences of all covariates were reduced to less than 0.1, indicating satisfactory post-matching balance. Specifically, the SMD of hospital scale decreased from 0.185 to 0.034, the number of beds from 0.142 to 0.027, and the number of medical staff from 0.158 to 0.041, compared with the pre-matching values. Figure 1 demonstrates more intuitively the standardized mean values of the propensity scores before and after matching. The results of the paired t-test showed a significant improvement in the balance of covariates after matching (*t* = 3.412, *p* = 0.025). These findings confirm the effectiveness of the matching procedure, which has effectively mitigated selection bias and laid a reliable foundation for subsequent empirical analyses.

**Table 2:** Covariate balance test table before and after PSM matching.

Covariate	Standardized difference before matching	Standardized difference after matching	Pre-matching *p*-value	Post-matching *p*-value
Hospital size	0.185	0.034	0.086	0.742
Number of beds	0.142	0.027	0.187	0.812
Medical staffing	0.158	0.041	0.143	0.697
Patient age composition	0.127	0.023	0.236	0.834
Regional economic level	0.103	0.018	0.347	0.879
Hospital type	0.094	0.025	0.398	0.823

### 3.2 Analysis of changes in revenue structure

**Figure 2:**
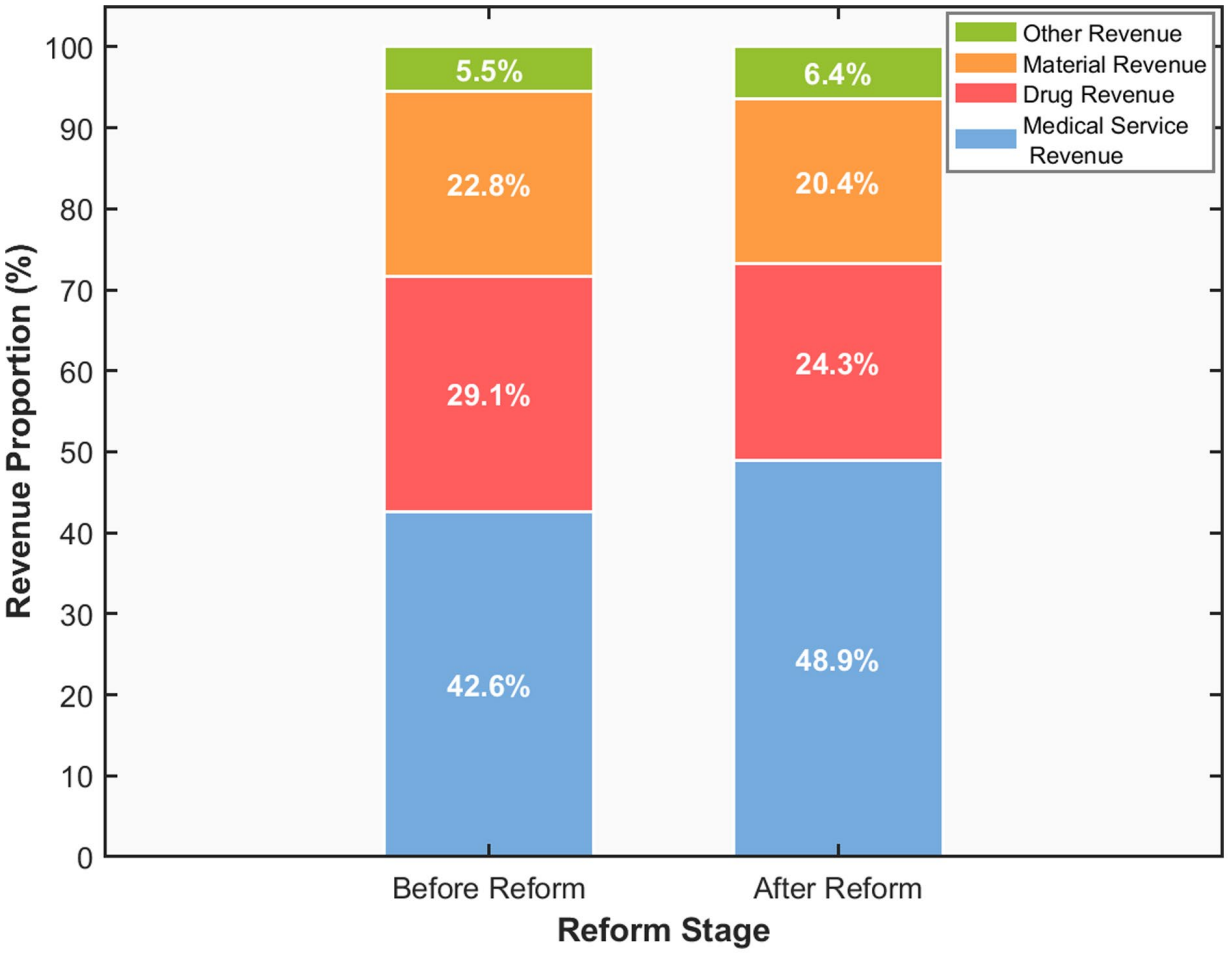
Hospital revenue structure changes before and after DRG reform.

The DRG payment reform has exerted a significant impact on the revenue structure of hospitals, with each revenue component exhibiting distinct adjustment trends (see Table 3). After the implementation of the reform, the average annual growth rate of total revenue of DRG-paying hospitals decreased from 11.8% before the reform to 8.4%, making revenue growth more rational and sustainable. The average annual growth rate of total revenue of control hospitals was 12.3% in the same period, and the difference between the two groups was statistically significant (*p* < 0.01). The restructuring of hospital revenue composition is shown in Table 3. Figure 2 provides a more intuitive visualization of the hospital revenue structure. Among DRG-paying hospitals, the proportion of medical service income increased from 42.6% before the reform to 48.9%, an increase of 6.3 percentage points. This shift reflects the rational realization of the value of medical professionals’ technical labor. Concurrently, the share of pharmaceutical revenue decreased significantly from 29.1% to 24.3%, a reduction of 4.8 percentage points (*p* < 0.001). The share of materials revenue decreased from 22.8% to 20.4%, a decrease of 2.4 percentage points (*p* < 0.01). The share of other revenues increased slightly from 5.5% to 6.4%. In contrast, control hospitals experienced more modest changes during the same period: medical service revenue share increased by only 0.8 percentage points (from 42.1% to 42.9%), while drug and material revenue shares decreased by 0.5 and 0.3 percentage points, respectively.

**Table 3:** Comparison of changes in hospital revenue structure before and after DRG reforms.

Income category	Before reform (%)	After reform (%)	Amount of change (percentage points)	*Z* value	*p*-value
Income from medical services	42.6	48.9	+6.3	8.524	<0.001
Drug revenue	29.1	24.3	−4.8	−7.892	<0.001
Material Revenue	22.8	20.4	−2.4	−5.673	<0.01
Other income	5.5	6.4	+0.9	2.145	0.032

To estimate the causal effect of DRG payment reform while controlling for underlying time trends and observable hospital characteristics, we applied the difference-in-differences regression model. Table 4 presents the formal DiD regression results for the revenue structure. The interaction term coefficient (Post × DRG, denoted as *β*_3_) captures the average treatment effect of DRG payment reform on revenue composition. The results show that, compared to control hospitals, DRG payment reform caused a statistically significant increase of 5.8 percentage points in the medical service revenue share (*β*_3_ = 0.058, SE = 0.012, *p* < 0.001), a decrease of 4.5 percentage points in drug revenue share (*β*_3_ = −0.045, SE = 0.011, p < 0.001), and a decrease of 2.3 percentage points in material revenue share (*β*_3_ = −0.023, SE = 0.009, *p* < 0.01). The coefficient for other revenue share was positive but smaller in magnitude (*β*_3_ = 0.010, SE = 0.005, *p* < 0.05). These DiD estimates represent the net policy effect after removing time-invariant differences between DRG-paying and control hospitals, as well as common temporal trends affecting all hospitals. The estimated treatment effects reflect the reasonable return of the value of medical technical labor and the effective control of dependence on drug and material revenues, consistent with the reform’s policy objectives.

**Table 4:** Difference-in-differences regression results for hospital revenue structure.

Variable	Income from medical services (%)	Drug revenue (%)	Material revenue (%)	Other revenue (%)
	(1)	(2)	(3)	(4)
Post (*β*₁)	0.008	−0.005	−0.003	0.001
	(0.008)	(0.007)	(0.005)	(0.003)
DRG (β₂)	0.005	−0.003	0.001	−0.002
	(0.014)	(0.012)	(0.009)	(0.005)
Post × DRG (β_3_)	0.058***	−0.045***	−0.023**	0.010*
	(0.012)	(0.011)	(0.009)	(0.005)
Control variables	Yes	Yes	Yes	Yes
Hospital fixed effects	Yes	Yes	Yes	Yes
Year fixed effects	Yes	Yes	Yes	Yes
*N*	336	336	336	336
*R* ^2^	0.842	0.798	0.765	0.623

Standard errors in parentheses are clustered at the hospital level to account for within-hospital correlation. ***p < 0.001, **p < 0.01, *p < 0.05. Post = 1 for years 2019–2023, 0 for years 2017–2018. DRG = 1 for DRG-paying hospitals (*n* = 24), 0 for control hospitals (*n* = 24). The interaction term coefficient (β_3_) represents the difference-in-differences estimate of the average treatment effect of DRG payment reform on revenue structure. Control variables include hospital scale (total revenue in 2017, in logarithms), number of beds, number of medical staff, average patient age, proportion of male patients, case mix index, and regional economic level (GDP per capita). *N* = 336 represents 48 hospitals × 7 years (2017–2023). The dependent variable is measured as the percentage share of each revenue component in total hospital revenue.

The departmental revenue analysis is shown in Table 5, where Cardiothoracic Surgery saw the most significant decrease in material revenue share from 33.2% to 27.8% due to the high complexity of the procedures and the high use of consumables. Neurosurgery had the largest increase in the proportion of medical service revenue, rising from 40.5% to 49.7%. General Surgery and Urology had relatively mild yet steadily advancing revenue restructuring, realizing a 5.2% and 4.8% increase in medical service revenue share, respectively.

**Table 5:** Analysis of changes in revenue structure by department.

Department	Income category	Before reform (%)	After reform (%)	Range of change (percentage points)	*t*-value	*p*-value
General Surgery	Medical service income	41.8	47.0	+5.2	6.234	<0.001
	Pharmaceutical revenue	30.2	25.8	−4.4	−5.891	<0.001
	Material revenue	22.1	20.3	−1.8	−3.456	0.001
	Other income	5.9	6.9	+1.0	2.123	0.035
Cardiothoracic surgery	Revenue from medical services	38.4	43.7	+5.3	5.789	<0.001
	Drug revenue	24.2	22.1	−2.1	−2.987	0.003
	Material revenue	33.2	27.8	−5.4	−7.234	<0.001
	Other income	4.2	6.4	+2.2	3.567	<0.001
Neurosurgery	Revenue from medical services	40.5	49.7	+9.2	8.945	<0.001
	Drug revenue	28.7	22.9	−5.8	−6.789	<0.001
	Material revenue	26.1	21.8	−4.3	−5.234	<0.001
	Other income	4.7	5.6	+0.9	1.876	0.062
Urology	Revenue from medical services	44.3	49.1	+4.8	5.432	<0.001
	Drug revenue	31.5	26.2	−5.3	−6.123	<0.001
	Material revenue	19.8	18.4	−1.4	−2.345	0.020
	Other income	4.4	6.3	+1.9	3.234	0.001

### 3.3 Analysis of changes in expenditure structure

**Figure 3:**
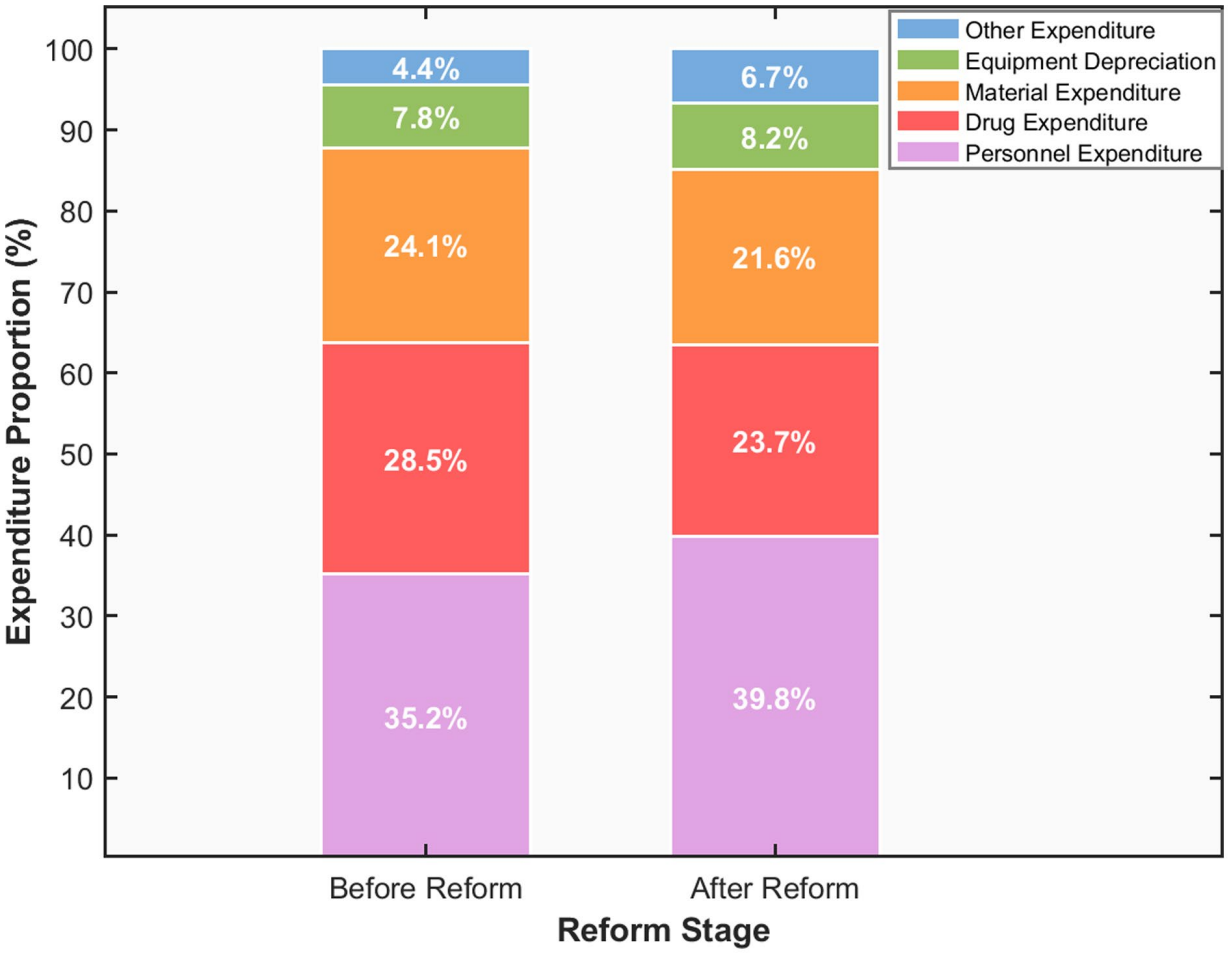
Hospital expenditure structure changes before and after DRG reform.

While adjusting the revenue structure of hospitals, the DRG payment reform has also exerted a significant optimizing effect on their expenditure structure. As presented in Table 6, the average annual expenditure growth rate of DRG-adopting hospitals declined from 12.5% to 7.8%, representing a decrease of 4.7 percentage points. In contrast, the average annual expenditure growth rate of control group hospitals only fell from 12.8% to 11.9% during the same period. The difference between the two groups is significant (*p* < 0.01). The assessment of the cost control effect shows that the DRG payment reform has reduced the average hospital expenditure level by 8.3%, with the most obvious control effect observed on drug and material expenditures. Figure 3 more intuitively reflects the structural changes in expenditures under the two payment systems. Among DRG-paying hospitals, the proportion of personnel expenditure increased from 35.2% before the reform to 39.8%, with an increase of 4.6 percentage points, reflecting the emphasis on the value of medical personnel and the improvement of incentive mechanisms. The proportion of pharmaceutical expenditure decreased significantly from 28.5% to 23.7%, representing a reduction of 4.8 percentage points (*p* < 0.001), which is highly consistent with the declining trend observed in the share of pharmaceutical revenue. Meanwhile, the proportion of medical material expenditure decreased by 2.5 percentage points, falling from 24.1% to 21.6% (*p* < 0.01). The share of equipment depreciation expenditure edged up from 7.8% to 8.2%, and the share of other expenditure increased from 4.4 to 6.7%, mainly reflecting the increase in management costs and quality control inputs. This adjustment in the expenditure structure indicates that the DRG payment system effectively guides hospitals to tilt resource allocation toward human capital while strictly controlling variable costs such as drugs and materials.

**Table 6:** Comparison of changes in hospital expenditure structure before and after the DRG reform.

Expenditure category	Before reform (%)	After reform (%)	Change amplitude (percentage point)	*Z* value	*p*-value
Personnel expenditure	35.2	39.8	+4.6	7.234	<0.001
Expenditure on medicines	28.5	23.7	−4.8	−8.156	<0.001
Material expenditure	24.1	21.6	−2.5	−4.892	<0.01
Depreciation of equipment	7.8	8.2	+0.4	1.567	0.117
Other expenditures	4.4	6.7	+2.3	4.123	<0.001

To formally test the causal effect of DRG payment reform on expenditure structure, we estimated the DiD regression model. Table 7 presents the regression results for expenditure composition. The DiD estimates show that, compared to control hospitals, DRG payment reform caused a statistically significant increase of 4.3 percentage points in personnel expenditure share (*β*_3_ = 0.043, SE = 0.013, *p* < 0.001), reflecting increased emphasis on the value of medical personnel and improved incentive mechanisms. The reform led to a decrease of 4.5 percentage points in drug expenditure share (*β*_3_ = −0.045, SE = 0.012, *p* < 0.001), which is highly consistent with the decreasing trend in drug revenue share, and a decrease of 2.4 percentage points in material expenditure share (*β*_3_ = −0.024, SE = 0.010, *p* < 0.01). Equipment depreciation expenditure share increased modestly (*β*_3_ = 0.006, SE = 0.004, *p* > 0.05), while other expenditure share increased by 2.0 percentage points (*β*_3_ = 0.020, SE = 0.008, *p* < 0.01). These regression-adjusted estimates confirm that DRG payment reform effectively guided hospitals to reallocate resources toward human capital while strictly controlling variable costs such as drugs and materials.

**Table 7:** Difference-in-differences regression results for hospital expenditure structure.

Variable	Personnel expenditure (%)	Expenditure on medicines (%)	Material expenditure (%)	Depreciation of equipment (%)	Other expenditures (%)
	(1)	(2)	(3)	(4)	(5)
Post (*β*_1_)	0.006	−0.007	−0.004	0.002	0.003
	(0.009)	(0.008)	(0.006)	(0.003)	(0.005)
DRG (*β*_2_)	0.004	−0.005	0.002	−0.001	0.000
	(0.016)	(0.014)	(0.011)	(0.005)	(0.007)
Post × DRG (*β*_3_)	0.043***	−0.045***	−0.024**	0.006	0.020**
	(0.013)	(0.012)	(0.010)	(0.004)	(0.008)
Control variables	Yes	Yes	Yes	Yes	Yes
Hospital fixed effects	Yes	Yes	Yes	Yes	Yes
Year fixed effects	Yes	Yes	Yes	Yes	Yes
*N*	336	336	336	336	336
*R* ^2^	0.856	0.812	0.778	0.691	0.645

**p < 0.01, ***p < 0.001.

Regarding overall cost control effectiveness, the average total expenditure level decreased by 8.3% among DRG-paying hospitals, compared with a 1.2% decrease in control hospitals, yielding a simple difference-in-differences calculation of 7.1 percentage points (*p* < 0.001). To account for observable hospital characteristics and time-varying confounders, we further estimated a DiD regression model controlling for hospital scale, staffing, patient composition, and regional economic factors, with hospital and year fixed effects. The regression-adjusted DiD estimate indicates that DRG payment reform reduced the total expenditure growth rate by 6.8 percentage points (*β*_3_ = −0.068, SE = 0.018, *p* < 0.001), slightly smaller than the simple DiD calculation but confirming robust cost control effects. This demonstrates that DRG payment significantly strengthened hospital cost containment relative to the traditional fee-for-service payment system, even after accounting for underlying differences between treatment and control hospitals.

### 3.4 Analysis of income and expenditure concentration

The DRG payment reform significantly improved the concentration and consistency of the distribution of hospital revenues and expenditures, as shown in Table 8. Results of the concentration index analysis indicate that the reform has achieved good standardization effects. The income concentration index increased from 0.342 pre-reform to 0.428, representing an increase of 25.1% (*p* < 0.001), and the expenditure concentration index increased from 0.356 to 0.441, an increase of 23.9% (*p* < 0.001). These findings suggest that the DRG payment system effectively mitigates abnormal fluctuations in income and expenditure levels among hospitals, thereby promoting the standardization of healthcare service provision.

**Table 8:** Comparison of changes in income and expenditure concentration index.

Concentration index	Before reform	After reform	Change amplitude (%)	*t*-value	*p*-value	95% confidence interval
Income concentration index	0.342	0.428	+25.1	8.456	<0.001	(0.065, 0.107)
Medical services revenue concentration	0.315	0.401	+27.3	7.234	<0.001	(0.062, 0.110).
Drug revenue Concentration	0.389	0.467	+20.1	6.789	<0.001	(0.052, 0.104)
Material Revenue Concentration	0.356	0.425	+19.4	5.892	<0.001	(0.043, 0.095)
Expenditure concentration index	0.356	0.441	+23.9	7.934	<0.001	(0.063, 0.107)
Concentration of personnel expenditures	0.298	0.372	+24.8	6.567	<0.001	(0.051, 0.097)
Concentration of drug expenditures	0.412	0.523	+26.9	8.123	<0.001	(0.079, 0.143)
Material Expenditure Concentration	0.367	0.448	+22.1	5.456	<0.001	(0.049, 0.113)
Composite concentration index	0.349	0.435	+24.6	9.234	<0.001	(0.068, 0.104)

Concentration index closer to 1 indicates a more concentrated distribution, and closer to 0 indicates a more dispersed distribution.

The cost dispersion analysis further validated the cost control effect of the reform, with the coefficient of variation of total cost decreasing from 0.247 before the reform to 0.189, representing a decrease of 23.5%. The coefficient of variation of drug costs decreased most significantly, from 0.312 to 0.203, a decrease of 34.9%, and the coefficient of variation of material costs decreased from 0.285 to 0.218, a decrease of 23.5%. The coefficient of variation of personnel costs increased slightly from 0.198 to 0.215, an increase of 8.6%, reflecting the differentiated strategies of different hospitals in human resource investment.

The assessment of resource allocation efficiency shows that the DRG payment reform has significantly improved hospital operational efficiency. The revenue-to-cost ratio improved from 1.082 before the reform to 1.136, representing an improvement of 5.0% (*p* < 0.01). Per capita revenue efficiency improved from 1.426 million yuan to 1.583 million yuan, an increase of 11.0%. Bed turnover improved from 1.34 times per year to 1.47 times per year, an improvement of 9.7%. The improvement of these indicators shows that the DRG payment system not only achieves the cost control goal but, more importantly, optimizes the efficiency of resource allocation and promotes the refinement and standardization of hospital management.

### 3.5 Parallel trend test and robustness analysis

**Figure 4:**
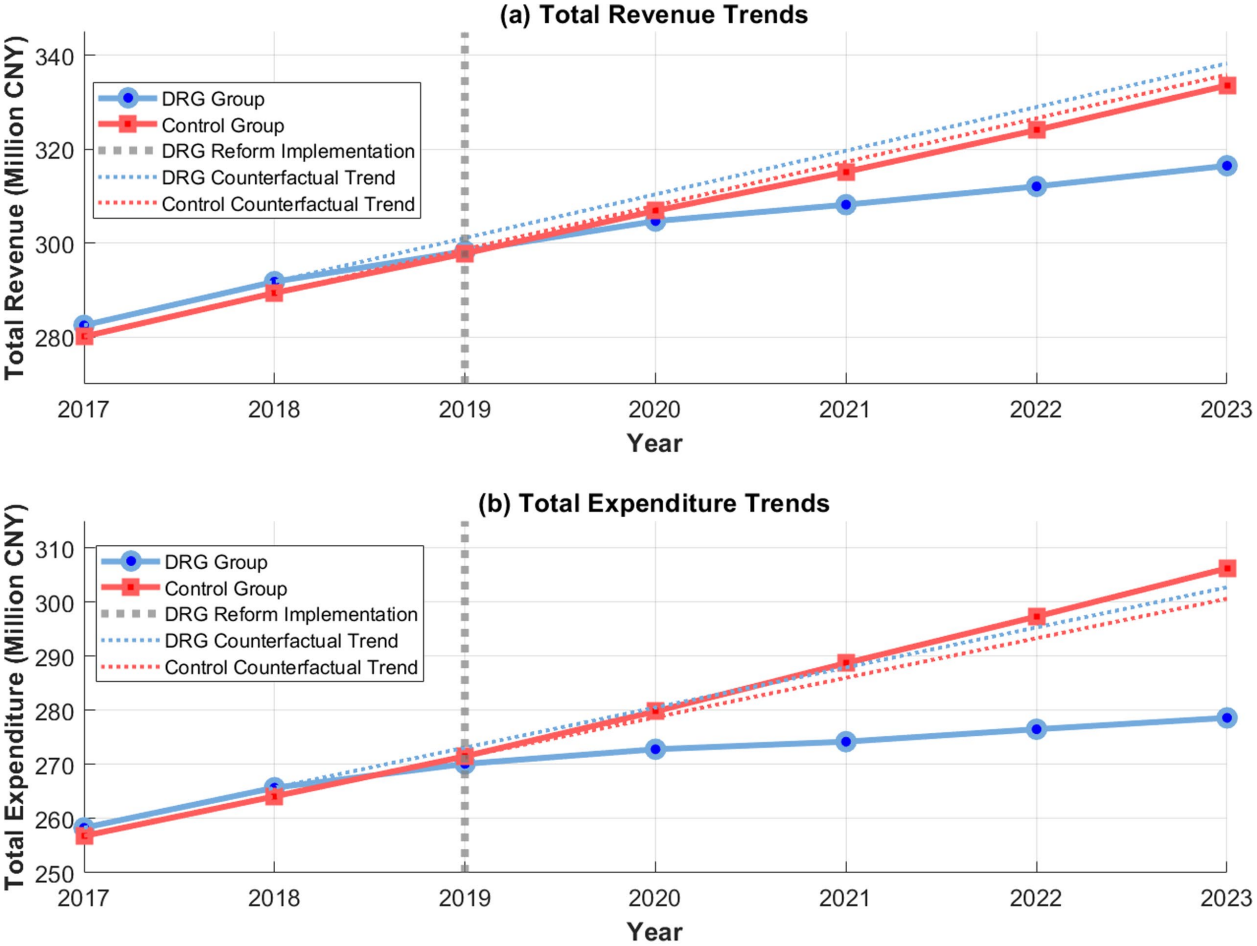
Parallel trend test: total revenue and expenditure.

To ensure the reliability of the estimation results of the double-difference model, the study conducted a rigorous test on the core assumptions of the model. The results of the parallel trend test are shown in Figure 4, which shows that the DRG payment group and the control group maintained a parallel trend in the income and expenditure structure indicators during the pre-reform period (2017**–**2018). The F-statistic for the parallel trend test of revenue structure is 1.234 (*p* = 0.298), and the F-statistic for the parallel trend test of expenditure structure is 0.987 (*p* = 0.376), both of which do not reach the level of significance, confirming the parallel trend hypothesis. The coefficients of the interaction term between the time dummy variable and the group dummy variable are not significant in all pre-reform years (*p* > 0.1), further verifying the validity of the model hypothesis.

The placebo test verifies the model’s robustness by randomly setting sham policy time points before the reform, and setting the sham reform time points as January 2018, June 2018, and December 2018. The test results show that the coefficients of the impact of the sham reform on the changes in the structure of income and expenditure are 0.023 (*p* = 0.654), −0.017 (*p* = 0.742), and 0.031 (*p* = 0.589), respectively. All coefficients are statistically insignificant, confirming that the observed effects of the actual DRG reform are not confounded by other unobserved factors, thereby validating the reliability of the core findings. Sensitivity analysis was conducted to verify the robustness of the results, employing different combinations of matching methods and control variables. The policy effect coefficients were −0.087 (*p* < 0.01) under the kernel matching method, and −0.092 (*p* < 0.01) under the Mahalanobis distance matching, which were basically the same as those in the baseline regression results. Furthermore, when adjusting the matching caliper to 0.01 and 0.1, respectively, the core research findings remained robust. And none of the confidence intervals for the policy effects contain zero, confirming the reliability of the findings.

## 4 Discussion

Based on the empirical analysis of the double-difference method, this study reveals the significant adjustment effect of the DRG payment reform on the income and expenditure structure of Chinese public hospitals. In terms of revenue structure, the proportion of medical service revenue increased from 42.6% pre-reform to 48.9%, representing an increase of 6.3 percentage points. This outcome fulfills the core reform objective of “restoring the value of medical services.” At the same time, the proportion of drug income dropped from 29.1% to 24.3%, and the proportion of material income dropped from 22.8% to 20.4%, effectively controlling the excessive reliance on drug and material income. The expenditure structure shows a reasonable adjustment trend, with the proportion of personnel expenditure increasing from 35.2% to 39.8%, reflecting the emphasis on the value of medical personnel. In contrast, the proportion of drug expenditure decreased from 28.5% to 23.7% and the proportion of material expenditure decreased from 24.1% to 21.6%, with a significant effect on cost control. A comparison with international experience shows that the results of this study are basically consistent with the effects of DRG reform in developed countries such as Germany (39) and Italy (40), which the reforms have achieved effective control of medical costs and optimization of resource allocation.

The DRG payment reform has reshaped the behavioral incentive mechanism of hospitals through a fundamental shift in the payment method. The paradigm change from pay-by-project to pay-by-disease has eliminated the possibility for hospitals to increase revenue by overutilizing pharmaceuticals and medical materials. Instead, under a fixed payment standard, hospitals must secure reasonable returns by enhancing operational efficiency and improving service quality (41). This payment constraint mechanism has directly promoted the transformation of hospitals from quantity-expansive development to quality-effective development, prompting hospitals to pay more attention to cost control and resource allocation optimization.

The reasons for the differences in the effectiveness of different departments mainly stem from the differences in surgical complexity and resource consumption patterns. Cardiothoracic Surgery, due to its high surgical complexity and high consumption of consumables, has seen the most significant decrease in the proportion of material income under the DRG payment constraint, from 33.2 to 27.8%, reflecting the strict control of the use of high-value consumables. Neurosurgery, as a technology-intensive department, witnessed the largest increase in the proportion of medical service income, rising from 40.5% to 49.7%. This change reflects the reasonable reflection of the value of technical labor. General surgery and urology, due to the relative standardization of diagnostic and treatment paths, are more easily adapted to the DRG payment model, and the revenue structure adjustment is relatively mild but sustained and stable.

The mechanism through which DRG payment may optimize resource allocation appears to lie in a gradual reorientation of incentives. By partly delinking hospital revenue from the use of drugs and materials and linking it more closely to service efficiency and quality, DRG payment is likely to encourage hospitals to allocate more resources to staff training, technological innovation, and service quality improvement, thereby supporting a shift from a predominantly factor-driven to a more efficiency-oriented development model. However, these positive effects do not mean that DRG-based payment is universally benign. International experience suggests that prospective payment systems can also generate unintended adverse incentives, including upcoding (classifying patients into higher-paying DRGs), premature discharge to shorten length of stay, and cream-skimming, whereby hospitals avoid complex or less profitable patients. The behaviors may undermine care quality, distort case mix, and shift costs to other parts of the system. As our study focuses on financial structures and efficiency outcomes, we do not directly observe changes in coding practices, readmissions, or patient severity. Future evaluations of DRG payment reform in China should therefore incorporate clinical quality indicators and patient-level data to monitor and mitigate these potential unintended consequences, such as by strengthening audit and feedback mechanisms, tracking risk-adjusted readmission rates, and refining DRG grouping and payment rules.

Based on the research findings, this study recommends further refining the policy design of the DRG payment system. Specifically, a differentiated payment standard system should be established, with standards calibrated to hospital grade, specialty characteristics, and disease complexity. This framework aims to ensure reasonable compensation for complex surgical procedures in tertiary hospitals while simultaneously enhancing the efficiency of resource utilization for standardized diseases. A dynamic adjustment mechanism encompassing real-time cost monitoring, regular data collection, and standardized evaluation and adjustment processes should be established. This mechanism aims to promptly identify deviations between DRG payment standards and actual medical costs, and implement targeted adjustments accordingly, thereby safeguarding the long-term effectiveness and adaptability of the DRG payment system. The synergistic promotion of supporting policies is crucial. Through the establishment of a salary distribution system compatible with DRG payment, the incorporation of cost control and quality indicators into the performance appraisal system, and the promotion of centralized procurement of medicines, policy synergies should be formed to provide comprehensive protection for the in-depth implementation of DRG payment reform.

Despite the rigorous quasi-experimental approach adopted in the methodological design and data analysis of this study, there are still some unavoidable limitations that need to be considered in the interpretation of the results. The representativeness of the sample data is somewhat limited. Although the sample of 48 tertiary public hospitals is statistically sufficient for testing efficacy, it mainly focuses on large-scale general hospitals, and its applicability to secondary hospitals, specialized hospitals, and hospitals in different regions may vary. This limitation, to a certain extent, affects the external validity of the research conclusions and the universality of the policy promotion. Moreover, although we used propensity score matching and hospital and year fixed effects to reduce observable differences between DRG and non-DRG hospitals, residual confounding due to unobserved institutional characteristics (e.g., management capacity or organizational culture) cannot be completely ruled out, and the estimated effects should therefore be interpreted as conservative approximations of the true causal impact of DRG reform.

Although our 7-year observation window captures medium-term changes, it is still insufficient to draw firm conclusions about the long-term financial sustainability of public hospitals. Moreover, financial sustainability depends not only on hospitals’ internal responses to DRG payment incentives but also on broader governance and financing arrangements, including hospital-level governance structures and external financial support such as government subsidies. Recent evidence from Greek public hospitals shows that state subsidies and delayed payments can mask underlying financial distress and shift risks to suppliers and the state owner, suggesting that standard financial ratios may overstate true financial viability (42). Experience from the COVID-19 emergency further illustrates how sudden shocks to elective activity and temporary compensation schemes can expose vulnerabilities in hospital business models and affect financial resilience (43). A more comprehensive assessment of whether DRG payment reform improves the financial sustainability of Chinese public hospitals will therefore require longer-term follow-up and explicit consideration of these governance and financing factors.

Despite the employment of propensity score matching and the difference-in-differences method to mitigate the impact of observable confounding factors, this study is still subject to certain limitations. Unobservable confounding variables, such as improvements in hospital management efficiency, adjustments to regional health policies, shifts in the healthcare market competitive landscape, and the disruptive effects of the pandemic, may potentially introduce biases into the research findings. The quality and completeness of hospital financial data may also vary, and inconsistencies in accounting standards and statistical calibers across hospitals may affect the comparability of data and the accuracy of analysis results. During 2020–2021, many tertiary public hospitals experienced reductions in elective surgeries and non-urgent admissions, which could mechanically reduce drug and material revenues and expenditures independent of DRG payment incentives. At the same time, additional staffing needs for infection prevention and control may have increased personnel costs. As a result, part of the observed changes in hospital revenue and expenditure structures may reflect pandemic-related shocks rather than the pure effect of DRG payment reform.

## 5 Conclusion

Based on a rigorous difference-in-differences analytical framework, this study systematically evaluates the effect of the DRG payment reform on the revenue and expenditure structure of Chinese public hospitals. The results of this study show that the DRG payment reform has effectively optimized the income and expenditure structure of hospitals, with the proportion of medical service income increasing from 42.6% to 48.9%, and the proportion of income from medicines and materials decreasing significantly. This outcome successfully realizes the core reform objective of “restoring the value of medical services.” The reform has significantly improved the efficiency of resource allocation, with the proportion of personnel expenditure reasonably increased to 39.8%, the revenue-to-cost ratio improved from 1.082 to 1.136, and the per capita revenue efficiency increased by 11.0%. These changes collectively reflect the transformation of hospitals from a factor-driven to an efficiency-driven development model. Additionally, the reform has increased the concentration of revenue and expenditure distribution by 23.5%, effectively mitigating abnormal fluctuations in economic performance across hospitals. The reform not only achieves the cost control goal but, more importantly, promotes the refinement and standardization of hospital management. The findings provide important empirical support for building a high-quality healthcare service system and deepening the reform of the healthcare payment system.

## Data Availability

The original contributions presented in the study are included in the article/supplementary material, further inquiries can be directed to the corresponding author.
